# Hypochlorous acid and hydrogen peroxide-induced negative regulation of *Salmonella enterica* serovar Typhimurium *ompW* by the response regulator ArcA

**DOI:** 10.1186/1471-2180-12-63

**Published:** 2012-05-22

**Authors:** Eduardo H Morales, Iván L Calderón, Bernardo Collao, Fernando Gil, Steffen Porwollik, Michael McClelland, Claudia P Saavedra

**Affiliations:** 1 Laboratorio de Microbiología Molecular, Facultad Ciencias Biológicas, Universidad Andres Bello, Santiago, Chile; 2 The Vaccine Research Institute of San Diego, 3030 Bunker Hill Suite# 203, San Diego, CA, 92109, USA; 3 Department of Pathology and Laboratory Medicine, D440 Medical Sciences I, University of California, Irvine, CA, 92697, USA

## Abstract

**Background:**

Hydrogen peroxide (H_2_O_2_) and hypochlorous acid (HOCl) are reactive oxygen species that are part of the oxidative burst encountered by *Salmonella enterica* serovar Typhimurium (*S*. Typhimurium) upon internalization by phagocytic cells. In order to survive, bacteria must sense these signals and modulate gene expression. Growing evidence indicates that the ArcAB two component system plays a role in the resistance to reactive oxygen species. We investigated the influx of H_2_O_2_ and HOCl through OmpW and the role of ArcAB in modulating its expression after exposure to both toxic compounds in *S.* Typhimurium.

**Results:**

H_2_O_2_ and HOCl influx was determined both *in vitro* and *in vivo*. A *S*. Typhimurium *ompW* mutant strain (∆*ompW*) exposed to sub-lethal levels of H_2_O_2_ and HOCl showed a decreased influx of both compounds as compared to a wild type strain. Further evidence of H_2_O_2_ and HOCl diffusion through OmpW was obtained by using reconstituted proteoliposomes. We hypothesized that *ompW* expression should be negatively regulated upon exposure to H_2_O_2_ and HOCl to better exclude these compounds from the cell. As expected, qRT-PCR showed a negative regulation in a wild type strain treated with sub-lethal concentrations of these compounds. A bioinformatic analysis in search for potential negative regulators predicted the presence of three ArcA binding sites at the *ompW* promoter region. By electrophoretic mobility shift assay (EMSA) and using transcriptional fusions we demonstrated an interaction between ArcA and one site at the *ompW* promoter region. Moreover, qRT-PCR showed that the negative regulation observed in the wild type strain was lost in an *arcA* and in *arcB* mutant strains.

**Conclusions:**

OmpW allows the influx of H_2_O_2_ and HOCl and is negatively regulated by ArcA by direct interaction with the *ompW* promoter region upon exposure to both toxic compounds.

## Background

Hydrogen peroxide (H_2_O_2_) and hypochlorous acid (HOCl) are reactive oxygen species that are part of the oxidative burst encountered by *S*. Typhimurium upon internalization by phagocytic cells. Under acidic conditions, such as those found inside the phagosome, H_2_O_2_ is generated spontaneously by the reaction of two superoxide anion (O_2_^−^) molecules [1]. Moreover, *S*. Typhimurium encodes both periplasmic and cytoplasmic superoxide dismutases that catalyze O_2_^−^ dismutation to generate H_2_O_2_ and molecular oxygen [2-4]. HOCl is produced by the action of myeloperoxidase (MPO) in a reaction that depends on H_2_O_2_, Cl^−^and acidic conditions [5,6]. Taken together, H_2_O_2_ and HOCl react with thiol and heme groups, copper and iron salts generating the reactive hydroxyl radical (OH^.^). As a consequence, they produce lipid peroxidation, chlorination of tyrosine residues, oxidation of iron centers, protein cross linking and DNA damage [5-8].

In order to enter Gram negative bacteria, H_2_O_2_ and HOCl must be able to cross the outer membrane (OM) and even though several biological membranes are permeable to H_2_O_2_, studies in *E. coli* and *Saccharomyces cerevisiae* showed that this compound cannot diffuse freely [9,10]. For HOCl, diffusion through the OM is reported to be limited [11]. One possibility for H_2_O_2_ and HOCl influx through the OM is diffusion through porins. In this context, we recently reported that OmpD, *S*. Typhimurium most abundant OM porin, allows H_2_O_2_ diffusion [12]. OM porins are organized as homo-trimers (classic porins) or monomers (small porins) forming aqueous channels that allow the influx of hydrophilic solutes with a molecular weight ≤ 600 Da [13]. Classic porins, including OmpC and OmpF, form β-barrels with 12–22 transmembrane segments while small porins (OmpW) are composed of 8–10 [14,15]. The crystal structure of OmpW from *E. coli* revealed that it forms an 8-stranded β-barrel and functions as an ion channel in lipid bilayers [16,17]. In *Vibrio cholerae*, OmpW was described as an immunogenic 22 KDa protein [18] and its expression is altered by factors such as temperature, salinity, nutrient availability and oxygen levels [19]. Additionally, several studies show that porins are regulated by ROS. Due its oxidant nature and diffusion through the OM, regulation of porin expression must be tightly regulated as a mechanism of controlling OM permeability. Accordingly, *S.* Typhimurium *ompD* and *ompW* expression is regulated in response to H_2_O_2_ and paraquat [12,20], respectively, and *S*. Enteritidis and Typhimurium exposure to HOCl results in lower levels of *ompDompC* and *ompF* transcripts [21].

The cellular response to oxidative stress is regulated at the transcriptional level by activating the SoxRS and OxyR regulons in response to O_2_^−^ and H_2_O_2_, respectively [22,23], however, several studies have provided evidence for a role of the ArcAB two component system in the resistance to ROS induced damage [12,24-26]. ArcA is essential for *S*. Enteritidis, Typhimurium and *E. coli* resistance to ROS [24,26,27]. ArcB is a sensor member of the histidine kinase family that is anchored to the inner membrane [28]. In response to oxygen availability, ArcB autophosphorylates in an ATP dependant intramolecular reaction at position His-292 [29,30] and transfers the phosphate group to the cytoplasmic response regulator ArcA [31-33], which binds to promoter regions regulating gene expression [34,35]. ArcB activity is regulated in response to oxygen conditions by the redox state of both the ubiquinone and menaquinone pools [29,36-38]. However, recent studies in *E. coli* show that the system is regulated by the degree of aerobiosis but not by the redox state of the ubiquinone pool, challenging the idea that the system is inhibited by oxidized quinones [39].

In this work we provide further evidence of the role of the ArcAB two component system in the response to ROS under aerobic conditions and show that this system mediates regulation of *ompW* expression in response to a novel signal, HOCl. First we demonstrate, both in *vivo* and *in vitro*, that OmpW mediates diffusion of H_2_O_2_ and HOCl and that exposure of *S*. Typhimurium to these compounds results in a negative regulation of *ompW*. By EMSA and using transcriptional fusions, we demonstrate that the global regulator ArcA binds to the *ompW* promoter region. Furthermore, we show that *ompW* negative regulation observed in wild type cells treated with H_2_O_2_ and HOCl was not retained in an *arcA* or *arcB* mutant strain, indicating that the ArcAB two component system mediates *ompW* negative regulation in response to H_2_O_2_ and HOCl. These results further expand our knowledge in both the mechanisms of ROS resistance and the role of ArcAB in this process.

## Results and discussion

### The OmpW porin facilitates H_2_O_2_ and HOCl diffusion through the OM and reconstituted proteoliposomes

Hydrogen peroxide and hypochlorous acid are ROS generated by phagocytic cells and in order to enter Gram-negative bacteria they must be able to cross the OM. Even though several biological membranes are permeable to H_2_O_2_, studies in *E. coli* and *S. cerevisiae* demonstrate that this compound cannot diffuse freely [9,10]. Additionally, the dielectric properties of H_2_O_2_ are comparable to those of water and this compound has a slighter larger dipolar moment, further limiting its diffusion through the OM lipid bilayer. For HOCl, diffusion through the OM is also reported to be limited [11]. Therefore, H_2_O_2_ and HOCl must be channeled through the lipid bilayer and one possibility is the influx through porins. We recently demonstrated that the most abundant OM protein in *S*. Typhimurium, OmpD, allows H_2_O_2_ diffusion and is regulated by ArcAB [12]. Little is known about the diffusion of HOCl, but genetic evidence has suggested that in *E. coli* porins might be used as entry channels for hypothiocyanate ions (OSCN^−^), a molecule with a similar chemical structure generated by lactoperoxidase using thiocyanate and H_2_O_2_ as an oxidant [40]. In one study, *ompC* and *ompF* knockout mutants showed an increased resistance to OSCN^−^, however, a direct role of porins in mediating HOCl diffusion was not evaluated.

**Figure 1:**
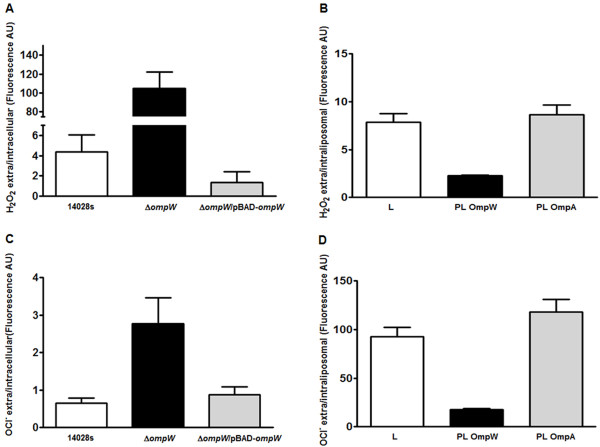
**OmpW facilitates H**_**2**_**O**_**2**_**and HOCl diffusion through the outer membrane and reconstituted proteoliposomes. A** and **C**. H_2_O_2_ and HOCl levels were measured indirectly by specific fluorescence assays in the wild type (14028s), mutant (∆*ompW*) and genetically complemented strains (∆*ompW*/pBAD-*ompW* + arabinose). Exponentially growing cells were exposed to H_2_O_2_**(A)** or NaOCl **(C)** for 5 min and fluorescence was determined in the extracellular (extra) and intracellular fractions. **B** and **D**. Free liposomes (L), proteoliposomes reconstituted with *S*. Typhimurium OmpW (PL OmpW) or OmpA (PL OmpA) proteins were incubated with H_2_O_2_**(B)** or NaOCl **(D)** for 5 min and fluorescence was determined in the extraliposomal (extra) and intraliposomal fractions. AU indicates arbitrary units. Values represent the average of four independent experiments ± SD.

To assess whether OmpW allows the diffusion of H_2_O_2_ and HOCl, scopoletin and dihydrorhodamine (DHR)-123 probes, respectively, were used to measure uptake of both toxic compounds separately in a wild type, ∆*ompW* and a genetically complemented ∆*ompW* (pBAD-*ompW*) strain as described in methods. The ∆*ompW* strain showed an increase in extracellular fluorescence levels after exposure to H_2_O_2_ and HOCl resulting in higher extra/intracellular ratios (24 and 4-fold, respectively) as compared to the wild type strain, indicating that in the absence of OmpW the influx of both toxic compounds is decreased. Genetic complementation of ∆*ompW* resulted in nearly identical levels of both extra and intracellular fluorescence as those observed in the wild type strain, suggesting that OmpW is necessary for H_2_O_2_ and HOCl uptake (Figure 1A and C). Even though OmpW appears as a direct responsible for the influx of the compounds, a pleiotropic effect cannot be ruled out at this point because the absence of OmpW in the mutant strain could be producing a remodeling of the membrane organization.

To establish a direct contribution of OmpW in H_2_O_2_ and HOCl transport, we used reconstituted proteoliposomes. OmpW-proteoliposomes showed a decrease in H_2_O_2_ and HOCl extra/intraliposomal ratios (3.5 and 5-fold respectively) when compared to free liposomes (Figure 1B and D). Proteoliposomes with *S*. Typhimurium OmpA porin were used as a negative control as previously described [12]. As expected, OmpA-proteoliposomes showed similar levels to those of free liposomes, indicating that OmpW facilitates H_2_O_2_ and HOCl uptake.

**Figure 2:**
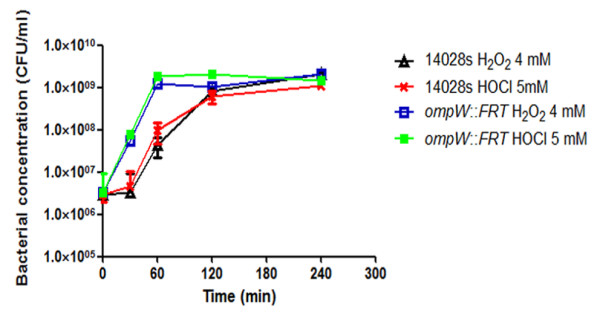
**Bacterial concentration of*S*. Typhimurium 14028s and Δ*ompW*exposed to H**_**2**_**O**_**2**_**or NaOCl.** Cultures of 14028s and Δ*ompW* were grown to OD ~ 0.4 and treated with H_2_O_2_ 4 mM or NaOCl 5 mM in LB medium. Time of treatment is indicated. Bacterial concentrations were determined by plating. The values are the concentrations of surviving bacteria after exposure to H_2_O_2_ or NaOCl. Experiments were performed in triplicate. Error bars indicate SD.

Since OmpW channels both toxic compounds across the lipid bilayer, we hypothesized that a ∆*ompW* strain should be more resistant to both toxic compounds when compared to the wild type strain. As shown in Figure 2, exposure of ∆*ompW* to H_2_O_2_ 4 mM or HOCl 5 mM resulted in an increase in the number of colony forming units (CFU) after 60 min of treatment. However, at longer periods the CFU count between strains 14028s and ∆*ompW* was similar. At 30 min post-treatment with either of the toxic compounds, strain ∆*ompW* showed an increase from 1×10^6^ CFU/ml to approximately 6×10^7^ CFU/ml. In contrast, the CFU/ml count for strain 14028s remained almost unaltered at 1×10^6^, resulting in a 1.5-log_10_-fold increase in growth for ∆*ompW*. A similar result was observed after 60 min of treatment where the *ompW* mutant strain showed an increase from 6×10^7^ to 1.5×10^9^ CFU/ml while the wild type strain changed from 1×10^6^ to 8×10^7^ CFU/ml. Our results suggest that the absence of OmpW in the mutant strain represents an advantage at short time points due to a decreased permeability towards both H_2_O_2_ and HOCl. At longer periods, OM permeability should be reduced because exposure to both toxic compounds results in a negative regulation of *S*. Typhimurium porins including OmpD, OmpC and OmpF [12,21]. One important possibility that cannot be ruled out at this time is that in the ∆*ompW* strain, the expression of other porins or the OM lipid composition might be altered, therefore changing OM permeability. For example, a study conducted in *E*. coli showed that an *ompC* knockout mutant had increased levels of OmpA [40], however, changes in permeability were not evaluated. Furthermore, this has not been evaluated in a *S*. Typhimurium or *E. coli* ∆*ompW* strain.

Our data supports the proposed model where OmpW allows the influx of small polar molecules, like H_2_O_2_ and HOCl. The crystal structure of OmpW from *E. coli* revealed that the cross-section of the barrel has approximate dimensions of 17 × 12 Å along the length of the barrel and although the interior of the channel has a hydrophobic character, the observed single channel activities shows that polar molecules traverse the barrel [17]. Taken together, these results provide biochemical and genetic evidence indicating that both toxic compounds are channeled through OmpW. From our knowledge, this is the first direct evidence of HOCl diffusion through porins. Furthermore, preliminary analyses indicate that H_2_O_2_ and HOCl channeling is common for *S*. Typhimurium OmpD, OmpC and OmpF porins (unpublished data).

### Hydrogen peroxide and hypochlorous acid exposure results in *ompW* negative regulation

**Figure 3:**
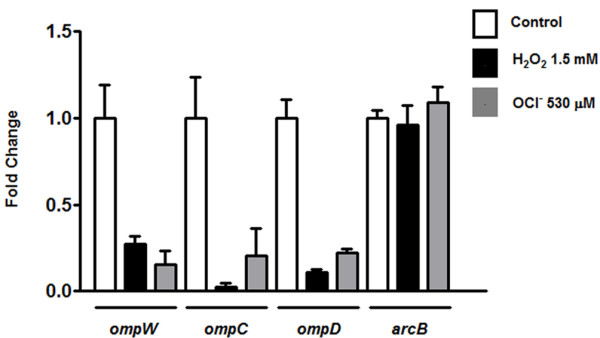
**Effect of H**_**2**_**O**_**2**_**and HOCl on*ompW*expression.** Wild type (14028s) exponentially growing cells were treated with H_2_O_2_ (1.5 mM) or NaOCl (530 μM) for 20 min and *ompW*, *ompD*, *ompC* and *arcB* mRNA levels were measured by qRT-PCR. Control cells received no treatment. 16S rRNA levels were used for normalization. Values represent the average of four independent experiments ± SD.

Since the OmpW porin channels H_2_O_2_ and HOCl through the OM and exposure to these molecules is detrimental to bacteria, we hypothesized that *ompW* should be negatively regulated when *S*. Typhimurium is exposed to H_2_O_2_ and HOCl. To study this effect, wild type *S*. Typhimurium cells were grown to mid-log phase, exposed to H_2_O_2_ or HOCl and *ompW* mRNA levels were measured by qRT-PCR. As seen in Figure 3, exposure to H_2_O_2_ and HOCl resulted in lower levels of *ompW* transcripts (0.27 ± 0.04 and 0.156 ± 0.079, respectively) relative to control untreated cells. In agreement with our results of *ompW* negative regulation, similar results were observed by Wang *et* al. (2010) who showed that *S.* Enteritidis and Typhimurium cells exposed to HOCl results in modulation of *ompD*, *ompC*, *ompF* (negatively) and *ompA* (positively) expression. Furthermore, Calderón *et* al. (2011) demonstrated that the *S*. Typhimurium *ompD* gene is negatively regulated in response to H_2_O_2_. Therefore, our and all the published data suggest that in the presence of OCl^-^ or H_2_O_2_ there might be a general lowering in the concentration of porins in the outer membrane, in order to diminish the permeability. To assess the specificity of our assay, we evaluated *ompD*, *ompC* and *arcB* transcript levels as positive (*ompD* and *ompC*) and negative controls (*arcB*). The *arcB* gene was used as a negative control based on our microarray analysis which shows that it remains unaltered under these conditions and between strains 1408s and Δ*arcA* (unpublished data). Our results indicate that after exposure to both toxic compounds, *arcB* transcript levels remain unchanged while those of *ompD* and *ompC* are lowered as compared to untreated cells (Figure 3). Therefore, all the evidence indicates that OM permeability is tightly regulated in response to ROS and could represent a novel mechanism of resistance when bacteria are exposed to these toxic compounds.

### ArcA binds the *ompW* promoter region

**Figure 4:**
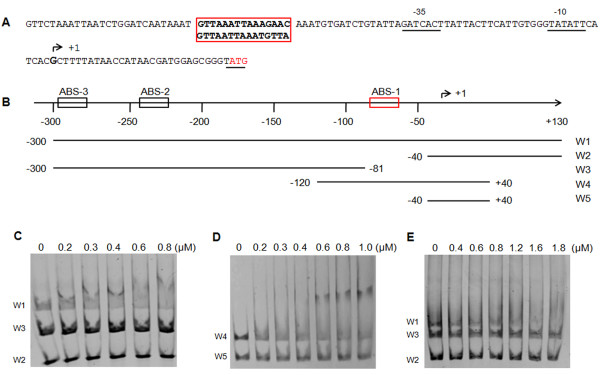
**ArcA binding to the*ompW*promoter region. A.***S*. Typhimurium *ompW* promoter region. Black and red boxes indicate predicted ArcA binding sites. -10 and −35 boxes are underlined. The transcription start site is shown in bold and indicated as +1. The translation start site is underlined and in red. The consensus ArcA binding site is shown under the promoter sequence. **B**. Schematic representation of the *ompW* promoter region. Positions relative to the transcription start site are indicated. ArcA binding sites are indicated as in the text. PCR products used in EMSAs are shown and names of each fragment are indicated. **C,D and E**. EMSA of the *ompW* promoter region. A 3-fold excess (60 ng) of fragments W2 and W3 were incubated with W1 **(C)** and the fragment W4 was incubated with W5 **(D)** and increasing amounts of phosphorylated ArcA as indicated on the top of each gel. **(E)** W1, W2 and W3 were incubated with increasing amounts of non-phosphorylated ArcA

In addition to the *soxRS* and *oxyR* systems, several studies have provided evidence that the ArcAB two component system plays an important role in the resistance to ROS induced damage. For example, ArcA is essential for *S*. Enteritidis and Typhimurium resistance to ROS [24,27] and *E. coli* mutant strains of the sensor ArcB and the regulator ArcA, show an increased susceptibility to H_2_O_2_[26]. However, neither of these studies identified genes directly regulated by the system under oxidative stress. We recently demonstrated that ArcA negatively regulates the expression of *S*. Typhimurium *ompD* after H_2_O_2_ exposure by direct interaction with its promoter region [12]. To determine if ArcA mediates *ompW* down-regulation in response to H_2_O_2_ and HOCl, a search for putative ArcA binding sites at the *ompW* promoter region was performed using Virtual Footprint 3.0 [41]. The analysis predicted the presence of three ArcA binding sites (ABS) located at positions −61 to −70 (ABS-1, forward orientation), -230 to −239 (ABS-2) and −286 to −295 (ABS-3, both in reverse orientation) relative to the experimentally determined transcription start site [42]. Comparison with the extended core region 5′-GTTAATTAAATGTTA-3′ described by Evans *et al*. (2011) further revealed that only ABS-1 presented a high degree of identity (14 out of 15 nucleotides) with the consensus sequence. To confirm or rule out a direct interaction between ArcA and the predicted binding sites, deletions of the promoter region were generated by PCR (schematized in Figure 4B) and used to perform non-radioactive EMSAs with ArcA and phosphorylated ArcA (ArcA-P). The purity of the protein was assessed by PAGE and ArcA was the dominant product. Electrophoretic mobility shift with ArcA-P was only observed when incubated with fragments that included ABS-1 (Figure 4C and D, W1 and W4). No shifts were observed in fragments that include both ABS-2 and ABS-3 (W3, even at three-fold excess) or control fragments that did not include any ABS (W2 and W5). Non-phosphorylated ArcA only generated electrophoretic mobility shifts at higher concentrations (over 1200 nM) where the negative controls were also retarded as a result of non-specific binding (Figure 3E). Taken together our bioinformatic and EMSA analyses indicate that ArcA-P binds to the *ompW* promoter region at a site located between positions −80 and -41 and suggests that this site is ABS-1 which is located between positions −70 to −55.

### Evaluating ArcA binding site 1 (ABS-1) functionality

**Figure 5:**
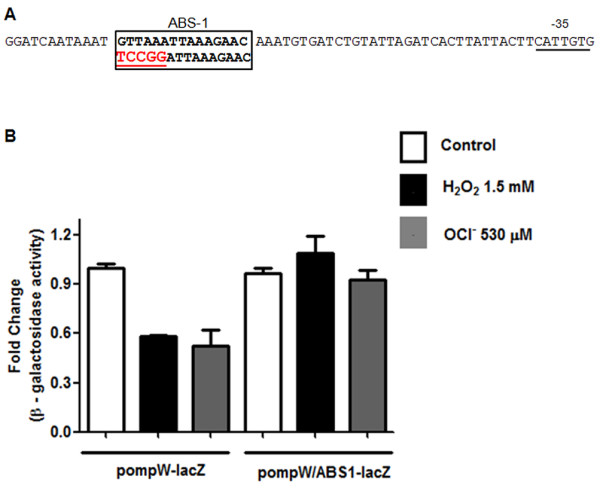
**Evaluating ArcA binding site 1 (ABS-1) functionality at the*ompW*promoter. (A)** Schematic representation of substitutions generated at the *ompW* promoter. Substituted bases are in red, underlined and shown below the core ArcA binding sequence. Black box indicates ABS-1. -35 is indicated. **(B)** Expression of the wild type and mutagenized regulatory region of *ompW* in *S*. Typhimurium. Strain 14028s was transformed with the reporter plasmids pompW-lacZ (wild type) or pompW/ABS1-lacZ (ABS-1 mutated). Cells were grown to OD ~ 0.4 and treated with H_2_O_2_ 1.5 mM or NaOCl 530 μM for 20 min and β-galactosidase activity was measured. Values represent the average of three independent experiments ± SD.

To further confirm that ABS-1 (Figure 4A) was the functional ArcA binding site mediating *ompW* negative regulation in response to ROS, we constructed transcriptional fusions of the *ompW* promoter region. We generated two different fusions which included the whole promoter from positions +1 to −600, with respect to the translation start site. One construction contained the native promoter (pompW-lacZ) while substitutions that mutated ABS-1 (shown in red and underlined, Figure 5A) were included in the second construction (pompW/ABS1-lacZ). The constructions were transformed into the wild type strain and β-galactosidase activity was measured in response to treatment with H_2_O_2_ and HOCl.

The activity of the constructions was compared to the untreated 14028s strain with the wild type fusion. Treatment of this strain with H_2_O_2_ and HOCl resulted in lower activity levels (0.58 ± 0.008 and 0.53 ± 0.095, respectively), in agreement with qRT-PCR experiments. However, a 5 nucleotide substitution of the most conserved residues at ABS-1 site (pompW/ABS1-lacZ) resulted in no regulation after exposure to either of the toxic compounds (1,09 ± 0.104 and 0,93 ± 0.061), indicating that they are relevant for the transcriptional activity of *ompW* in response to H_2_O_2_ and HOCl (Figure 5B). Furthermore, these results are in agreement with EMSAs which indicate that ArcA only binds to fragments containing ABS-1.

### The ArcAB two component system mediates *ompW* negative regulation

**Figure 6:**
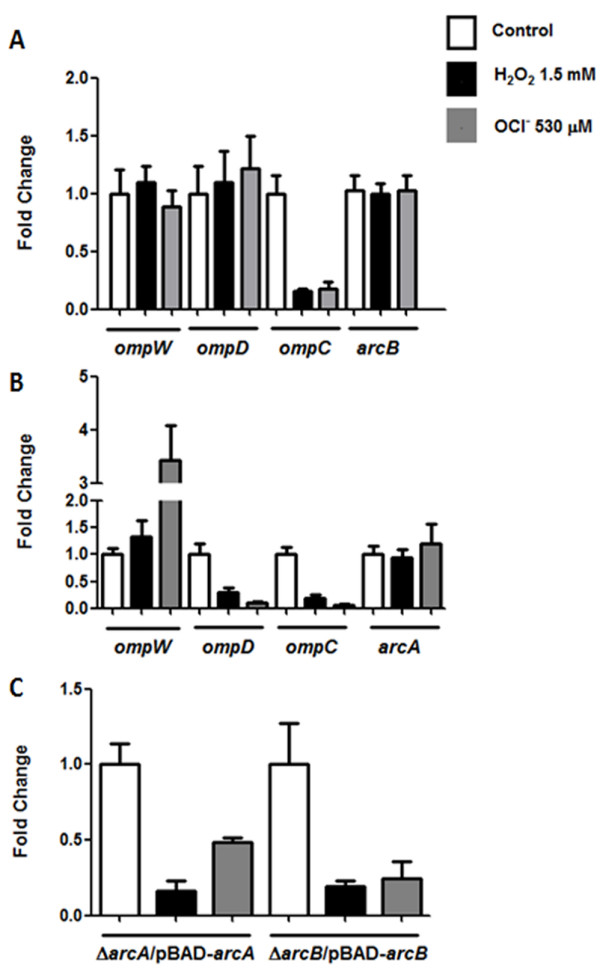
**ArcAB-dependant expression of*ompW*.***ompW*, *ompD*, *ompC*, *arcB* and *arcA* mRNA levels were measured by qRT-PCR in a **(A)** ∆*arcA*, **(B)** ∆*arcB* and **(C)** ∆*arcA*/pBAD-*arcA* and ∆*arcB*/pBAD-*arcB*. *arcB* and *arcA* were used as negative controls in **(A)** and **(B)**, respectively. Exponentially growing cells were treated with H_2_O_2_ 1.5 mM or NaOCl 530 μM for 20 min and transcript levels were measured. Genetically complemented cells were grown in the presence of arabinose 1 mM. Control cells received no treatment. 16S rRNA levels were used for normalization. Values represent the average of three independent experiments ± SD.

To establish a direct relationship between *ompW* negative regulation and ArcA-P binding to its promoter region, *ompW* expression was evaluated by qRT-PCR in a ∆*arcA* strain exposed to H_2_O_2_ and HOCl. The negative regulation observed in the wild type strain was not retained in an *arcA* mutant treated with either of the toxic compounds and *ompW* transcript levels were similar as those observed in untreated cells. Genetic complementation of ∆*arcA* restored the negative regulation observed in wild type cells exhibiting lower *ompW* mRNA levels (0.161 ± 0.068 and 0.488 ± 0.027, respectively) as compared to untreated cells (Figure 6A and C). Growth of the genetically complemented strain in the presence of glucose (non-induction) resulted in similar *ompW* mRNA levels between treated and untreated cells (data not shown). As controls, we measured *ompDompC* and *arcB* transcript levels after exposure to H_2_O_2_ and HOCl in a ∆*arcA* strain. Transcript levels of *ompD* were measured since its expression is regulated by ArcA under ROS conditions [12]. Our results indicate that neither *ompD* or *arcB* transcript levels were decreased after exposure to H_2_O_2_ or HOCl while those of *ompC* remained regulated in a ∆*arcA* strain treated with either of the toxic compounds (Figure 6A), confirming that ArcA mediates *ompD* regulation under ROS conditions and showing that the expression of *ompC* is ArcA independent and regulated by different mechanisms which remain unsolved to the date, and are under study in our laboratory. Furthermore, our bioinformatic analyses in search for ArcA motifs predicted binding sites in the promoter regions of *ompW* and *ompD*, but not for *ompC* ([12], data not shown).

To determine whether the negative regulation by ArcA was dependant on its cognate sensor ArcB, *ompW* mRNA levels were evaluated in a ∆*arcB* strain. In contrast to the negative regulation observed in wild type cells, *ompW* mRNA levels were further increased in a ∆*arcB* strain after exposure to HOCl (3.37 ± 1.09). Transcript levels after treatment with H_2_O_2_ were similar as those observed in untreated cells (Figure 6B). One possibility for this result is that in the absence of ArcA, ArcB might phosphorylate (*i.e* ArcB-OmpR, [43]) one or more response regulators, either unspecifically or due to cross-talk, which could bind to the promoter region and therefore prevent binding of positive regulators like SoxS, which has been demonstrated to regulate *ompW* and is up-regulated in response to HOCl [20,44]. This could result in constant *ompW* transcript levels as shown in Figure 6A. On the other hand, in the absence of ArcB no phosphorylation occurs and SoxS or other positive regulator(s) might have free accessibility to the *ompW* promoter and therefore increase its expression (Figure 6B), although this possibility has not been evaluated in this study. Genetic complementation of ∆*arcB* restored the negative regulation observed in wild type cells exposed to H_2_O_2_ and HOCl (0.19 ± 0.04 and 0.24 ± 0.11, respectively, Figure 6C). The *ompD* and *ompC* transcripts levels remained down-regulated after exposure to H_2_O_2_ and HOCl in the ∆*arcB* strain, while the negative control *arcA* remained unaltered (Figure 6B).

The ArcA regulon in anaerobically grown *S*. Typhimurium was recently determined [27]. Interestingly, neither *ompD* nor *ompW* expression was down-regulated in an ArcA dependant manner, suggesting that the ArcA regulon under anaerobic and aerobic ROS conditions could be different. Even in *E. coliompW* expression is suggested to be regulated by FNR in response to oxygen availability [39]. The difference between the ArcA regulons under aerobic and ROS conditions might be explained by studies suggesting that the mechanism of ArcA activation under aerobic conditions is different from those classically described. *E. coli* mutant strains in residue H-717 of ArcB are able to phosphorylate and activate ArcA through the transfer of the phosphate group from residue His-292 under aerobic conditions [45] and Loui *et* al. (2009) suggested that H_2_O_2_ resistance is independent of ArcA phosphorylation at residue Asp-54. To the date, the detailed molecular mechanism of ArcAB activation in response to ROS remains unsolved. Therefore, further experiments to unveil the molecular mechanism by which the *S*. Typhimurium ArcAB two component system is activated are needed and under way in our laboratory.

## Conclusion

We provide both genetic and biochemical evidence indicating that the OM porin OmpW mediates the influx of H_2_O_2_ and HOCl. The results revealed that the *S*. Typhimurium *ompW* gene is negatively regulated upon exposure to both toxic compounds. Furthermore, we demonstrate that the response regulator ArcA mediates *ompW* negative regulation in response to H_2_O_2_ and HOCl via a direct interaction with the upstream region of *ompW*. Taken together, with our previous observation that OmpD mediates influx of H_2_O_2_ and is negatively regulated by ArcA in response to H_2_O_2_, these results further expand our knowledge regarding the coordinated regulatory mechanisms of ROS resistance and the role of ArcAB in this process.

## Methods

### Bacterial strains and growth conditions

Bacterial strains used in this work are listed in Table 1. Cells were grown aerobically with agitation in LB medium at 37°C. Solid media consisted of agar (20 g l^−1^) and plates were incubated at 37°C. Dilutions (1:100) of overnight cultures were used to initiate growth. When necessary, growth media was supplemented with the appropriate antibiotics (see below).

**Table 1:** Bacterial strains used in this study

Strain	Relevant characteristic(s)	Source
*S*. Typhimurium		
14028s	wild type strain	G. Mora
14028s/pompW-lacZ	14028s transformed with a derivative of plasmid pLacZ-Basic carrying the *ompW* promoter (nt −600 to +1)	This work
14028s/pompW/ABS1-lacZ	14028s transformed with a derivative of plasmid pLacZ-Basic carrying the *ompW* promoter (nt −600 to +1) with substitution GTTAA to TCCGG into position −70 to −66	This work
Δ*ompW*	*ompW*::*kan*	C. Saavedra
Δ*ompW/*pBAD-*ompW*	Δ*ompW* strain complemented with pBAD vector carrying the *S*. Typhimurium *ompW* gene	C. Saavedra
Δ*arcA*	*arcA*::*cam*	[12]
Δ*arcA/* pBAD-*arcA*	Δ*arcA* strain complemented with pBAD vector carrying the *S*. Typhimurium *arcA* gene	[12]
Δ*arcB*	*arcB*::*cam*	This work
Δ*arcB/* pBAD-*arcB*	Δ*arcB* strain complemented with pBAD vector carrying the *S*. Typhimurium *arcB* gene	This work
*E. coli* Top10	F- *mcrA* Δ(*mrr-hsd*RMS-*mcr*BC) Φ80*lac*ZΔM15 Δ*lac*Χ74 *rec*A1 *ara*D139 Δ(*ara-leu*)7697 *gal*U *gal*K *rps*L (StrR) *end*A1 *nup*G	Invitrogen
Top10 pBAD-*ompW*	Top10 transformed with the pBAD vector carrying the *S*. Typhimurium *ompW* gene	C. Saavedra
Top10 pBAD-*ompA*	Top10 transformed with the pBAD vector carrying the *S*. Typhimurium *ompA* gene	C. Saavedra
Top10 pBAD-*arcB*	Top10 transformed with the pBAD vector carrying the *S*. Typhimurium *arcB* gene	This work
BL21 pET-TOPOArcA	BL21(DE3) transformed with the pET-TOPO101ArcA vector carrying the *S*. Typhimurium *arcA* gene	[12]

### Strain construction and genetic complementation

*S*. Typhimurium *arcB* gene was interrupted by gene disruption as previously described [46]. Strain 14028s (wild type) harboring plasmid pKD46 was grown in the presence of arabinose (10 mM) and ampicillin (100 μg ml^−1^) to OD_600_ ~ 0.4, made electrocompetent and transformed with a PCR product generated with plasmid pKD3 as template and primers 5′ ATTGGGTATTATGTGCGAAGTTGTGGTGAAGGAATCCTCTTGTAGGCTGGAGCTGCTTCG 3′ (WarcBF) and 5′ GGTGTTGGCGCAGTATTCGCGCACCCCGGTCAAACCGGGGCATATGAATATCCTCCTTAG 3′ (WarcBR). Transformants were selected on LB plates supplemented with chloramphenicol (20 μg ml^−1^) and confirmed by PCR using primers 5′ GCTACGCATATTTCGCACAA 3′ (arcBF) and 5′ GCGCCTTTGACATCATCATA 3′ (arcBR).

Genetic complementation of the ∆*arcB* strain was performed using plasmid pBAD-*arcB*. To generate this plasmid, *S*. Typhimurium *arcB* gene was amplified by PCR using primers 5′ ATGAAGCAAATTCGTATGCTG 3′ (pBADarcBF) and 5′ TCATTTTTTTTCCGCGTTTGCCACCC 3′ (pBADarcBR) and cloned into plasmid pBAD-TOPO TA® (Invitrogen) according to manufacturer’s instructions. Insertion was verified by DNA sequencing.

### Bacterial survival after exposure to oxidative stress

Bacteria were cultured in 5 ml of LB medium at 37°C overnight with shaking. Antibiotics were added as appropriate. 1:1000 dilutions of the overnight cultures were grown in 25 ml to OD ~ 0.4 and H_2_O_2_ 4 mM or NaOCl 5 mM (final concentration) were added. In all the assays the cultures were grown aerobically at 250 rpm. Aliquots of cultures were withdrawn at the different time points, diluted and plated in triplicate. Bacterial cultures were enumerated by counting the number of CFU after overnight incubation to determine the bacterial concentrations.

### Construction of transcriptional fusions with reporter gene *lacZ*

The native *ompW* promoter region from positions +1 to −600 (with respect to the translation start) site was amplified by PCR with primers ompW_pLacZ_-600F_ATG 5′ CGGGGTACCCCCGATATCGAAAATTCGCG 3′ and ompW_pLacZ_-1R_ATG 5′ CCCAAGCTTACCCGCTCCATCGTTATGGT 3′ using genomic DNA from *S*. Typhimurium (strain 14028s). The restriction sites (K*pn*I and H*in*dIII, respectively) at the ends of the DNA fragment were introduced by the PCR primers (underlined sequences) and digested with the corresponding enzymes. The digested PCR product was cloned into the multiple cloning site (MCS) of the β-galactosidase reporter vector pLacZ-Basic (GenBank accession no. U13184), Clontech, generating plasmid pompW-lacZ. To generate plasmid pompW/ABS1-lacZ, primers ompW_pLacZ_-600F_ATG with Mut_sit_arcAR 5′ TGTTCTTATAATGCGGAATTTATTGATCCAG 3′ and ompW_pLacZ_-1R_ATG with Mut_sit_arcAF 5′ CTGGATCAATAAATTCCGGAATTATAAGAACA 3′ were used to generate overlapping PCR products spanning the whole length of the *ompW* promoter. Mutation of ABS-1 was generated by incorporating substitutions in primers Mut_sit_arcAF and Mut_sit_arcAR (underlined sequences). The resulting PCR products were used as templates in a second reaction with primers ompW_pLacZ_-600F_ATG and ompW_pLacZ_-1R_ATG to generate the mutated *ompW* promoter, which was digested and cloned into the MCS of plasmid pLacZ-Basic. Constructions were confirmed by DNA sequencing. The generated constructs were transformed into wild type strain 14028s. To evaluate activity, cells at OD_600_ ~ 0.4 were grown for 20 min in the presence of H_2_O_2_ (1.5 mM) or NaOCl (530 μM). Control cells received no treatment. β-galactosidase activity was determined as previously described [20].

### Protein purification

His-tagged ArcA used in EMSAs was purified as previously described [12]. Briefly *E. coli* BL21 cells harboring plasmid pET-TOPO-*arcA* were grown in 500 ml of LB medium supplemented with amplicillin (100 μg ml^−1^) to OD_600_ ~ 0.4 and protein overexpression was carried out by adding 1 mM IPTG and further growth for 6 h. Protein was purified by affinity chromatography as described by Georgellis *et* al., (1997).

Outer membrane proteins used in proteoliposomes were purified as described by Calderón *et* al. (2011). *E. coli* Top10 cells carrying pBAD-*ompA* or pBAD-*ompW* were grown in 500 ml to OD_600_ ~ 0.6 at 37°C and overexpression was performed for 5 h in the presence of 1 mM arabinose. His-tagged porins were purified by affinity chromatography using HisTrap HP columns (Amersham) according to the manufacturer’s instructions.

Plasmid pBAD-*ompW* was generated amplifying the coding region of *S*. Typhimurium *ompW* by PCR using primers 5′ ATGAAAAAATTTACAGTGGC 3′ (pBAD-ompWF) and 5′ GAAACGATAGCCTGCCGAG 3′ (pBAD-ompWR) and cloned into plasmid pBAD-TOPO TA® (Invitrogen) according to the manufacturer’s instructions. Insertion was verified by DNA sequencing.

### RNA isolation and *ompW* mRNA detection

Overnight cultures were diluted (1:100) and cells were grown to OD_600_ ~ 0.4. Genetically complemented cells (∆*arcA*/pBAD-*arcA* and ∆*arcB*/pBAD-*arcB*) were grown in the presence of arabinose (1 mM) and ampicillin (100 μg ml^-1^). At this point, H_2_O_2_ (1.5 mM) or NaOCl (530 μM) was added and cells were grown for 20 min. Control cells received no treatment. After exposure to the toxic compounds, 4 ml were withdrawn from the culture and used to extract total RNA using GenElute Total RNA purification Kit® (Sigma). Total RNA treatment with DNase I and cDNA synthesis was performed as previously described [19].

Relative quantification of *ompW* mRNA was performed using Brilliant II SYBR Green QPCR Master Reagent Kit and the Mx3000P detection system (Stratagene). 16S rRNA was used for normalization. Specific primers were 5′ ATGAAAAAATTTACAGTGG 3′ (RTompWF) and 5′ GAAACGATAGCCTGCCGA 3′ (RTompWR) for the *ompW* gene; 5′ GTAGAATTCCAGGTGTAGCG 3′ (16SF) and 5′ TTATCACTGGCAGTCTCCTT 3′ (16SR) for 16S rRNA gene (16S). The reaction mixture was carried out in a final volume of 20 μl containing 1 μl of diluted cDNA (1:1000), 0.24 μl of each primer (120 nM), 10 μl of 10 x Master Mix, 0.14 μl of diluted ROX (1:200) and 8.38 μl of H_2_O. The reaction was performed under the following conditions: 10 min at 95°C followed by 40 cycles of 30 s at 95°C, 30 s at 53°C and 45 s at 72°C. Finally a melting cycle from 53 to 95°C was performed to check for amplification specificity. Amplification efficiency was calculated from a standard curve constructed by amplifying serial dilutions of RT-PCR products for each gene. These values were used to obtain the fold change in expression for the gene of interest normalized with 16S levels according to [47]. Experiments were performed in three biological and technical replicates.

### DNA binding assays

Non-radioactive EMSAs were performed as described [48]. Briefly, increasing amounts of purified ArcA (phosphorylated and unphosphorylated) were incubated with 20 or 60 ng of PCR product(s) in binding buffer (100 mM Tris-Cl [pH 7.4], 100 mM KCl, 10 mM MgCl_2_, 10% glycerol, and 2 mM dithiothreitol) for 20 min at 30°C. Reaction mixtures were immediately loaded on prerun 4% native polyacrylamide gels. The DNA-protein complexes were visualized by ethidium bromide staining. PCR fragments used in EMSAs were generated by PCR using reverse primer 5′ ACCCGCTCCATCGTTATGGT 3′ (ompWR) in combination with 5′ GAGCAGACAAATATTTGCAT 3′ (300WF) or 5′ TATTAGATCACTTATTACTT 3′ (170WF) to generate fragments W1 and W2, respectively. Fragment W3 was generated using primers 300WF and 5′ GATCCAGATTAATTTAGAAC 3′. Fragments W4 and W5 were generated by using reverse primer 5′ AATTTTTTCATACCCGCTCC 3′ in combination with primers 5′ CCTATAACCAGGATTTTCAA 3′ and 170WF, respectively. ArcA phosphorylation was carried out as described by Linch and Lin (1996). Briefly purified ArcA was incubated with 50 mM disodium carbamoyl phosphate (Sigma) in a buffer containing 100 mM Tris-Cl (pH 7.4), 10 mM MgCl_2_, 125 mM KCl, for 1 h at 30°C and used immediately in EMSA assays.

### *In vivo* and *in vitro* determination of hydrogen peroxide and hypochlorous acid diffusion

*In vivo* diffusion of H_2_O_2_ was assessed as previously described [12]. For HOCl detection, overnight cultures were diluted and cells were grown to OD_600_ ~ 0.5. Two ml of bacterial culture were centrifuged for 5 min at 4500 x *g* and resuspended in 1 ml of 100 mM phosphate buffer (pH 7.2). A 200 μl aliquot was incubated for 5 min with 530 μM NaOCl and constant agitation. Following, cells were vacuum filtered using polycarbonate filters of 0.025 μm (Millipore) and pass through was collected (extracellular fraction). Bacteria retained in the filter were recovered with 1 ml of 50 mM phosphate buffer (pH 7.2) and disrupted by sonication (intracellular fraction). Both fractions (190 μl) were incubated separately with dihydrorhodamine-123 to a final concentration of 5 μM as previously described [49]. The fluorescent product, rhodamine-123, was measured by fluorescence detection with excitation and emission wavelengths of 500 and 536 nm, respectively. HOCl and H_2_O_2_ uptake was determined as the extracellular/intracellular fluorescence ratio. The background fluorescence from a bacterial suspension not exposed to either of the toxic compounds was subtracted and results were normalized by protein concentration.

Proteoliposomes were prepared as described [50] with modifications [51]. For *in vitro* diffusion, proteoliposomes were incubated with 1.5 mM H_2_O_2_ or 530 μM NaOCl for 5 min, vacuum filtered and pass through was recovered (extraliposomal fraction). Proteoliposomes were recovered from the filters with 2 ml of 50 mM phosphate buffer (pH 7.2) and disrupted by sonication (intraliposomal fraction). Fluorescence was measured in both fractions as described above and H_2_O_2_ or HOCl uptake was determined as the extraliposomal/intraliposomal fluorescence ratio.

## Misc

Eduardo H Morales and Iván L Calderón contributed equally to this work

## Author’s contributions

EHM and CPS conceived the project. EHM, BC and ILC performed the experiments. FG and SPo conducted partial data analysis. EHM, ILC, MM and CPS wrote the paper. All authors read and approved the final manuscript.
